# A quality improvement project to increase compliance with diabetes measures in an academic outpatient setting

**DOI:** 10.1186/s40842-019-0084-9

**Published:** 2019-07-23

**Authors:** Subhash Edupuganti, Jordan Bushman, Rhyan Maditz, Pradeep Kaminoulu, Alexandra Halalau

**Affiliations:** 1 Oakland University William Beaumont School of Medicine, 3601 W 13 Mile Rd, Royal Oak, Michigan, Rochester Hills, MI 48073 USA; 2 Department of Internal Medicine, William Beaumont Hospital, Royal Oak, MI USA; 3 Department of Nephrology, Cleveland Clinic Foundation, Cleveland, OH USA

**Keywords:** Quality improvement, Diabetes mellitus, Resident clinic, Diabetes preventative care

## Abstract

**Background:**

American Diabetes Association (ADA) sets annual guidelines on preventative measures that aim to delay the onset of severe diabetes mellitus complications. Compared to private internal medicine clinics, resident clinics provide suboptimal diabetic preventative care as evidenced by decreased compliance with ADA guidelines. The purpose of our study is to improve diabetic care in resident clinics through quality improvement (QI) projects, with A1C value as primary outcome and other ADA guidelines as secondary outcomes.

**Methods:**

Our resident clinic at Beaumont Hospital, Royal Oak consists of 76 residents divided in 8 teams. In November 2016, baseline data on ADA guideline measures was obtained on 538 patients with diabetes mellitus. A root cause analysis was conducted. 5 teams developed a QI intervention plan to improve their diabetes care and 3 teams served as comparisons without intervention plans. In November 2017, post-intervention data was collected.

**Results:**

Baseline characteristics demonstrate mean age of intervention groups at 60.9 years and of comparison groups at 58.9 years. The change in A1C value from baseline to post-intervention was + 0.09 vs. + 0.322 in the intervention and comparison groups respectively (*p* = 0.174). As a group, the changes in secondary outcome measures were as follows: eye examinations (+ 5% in intervention vs. -7% in comparison, *p* < 0.01), foot examinations (+ 13% vs. + 5%, *p* = 0.09), lipid panel testing (+ 7% vs. -5%, *p* < 0.01), micro-albumin/creatinine ratio testing (+ 4% vs. + 1%, *p* = 0.03), and A1C testing (+8% vs. + 5%, *p* = 0.24).

**Conclusions:**

While the QI project did not improve A1C value, it did have significant improvement in several secondary outcomes within intervention groups. One resident team implemented an intervention involving protected half-day blocks to identify overdue examinations and consequently had the largest improvements, thus serving as a potential intervention to further study. Given our study results, we believe that QI interventions improve preventative care for patients with diabetes in resident clinics.

**Electronic supplementary material:**

The online version of this article (10.1186/s40842-019-0084-9) contains supplementary material, which is available to authorized users.

## Background

Diabetes mellitus is one of the most common chronic diseases in the United States, with a prevalence of an estimated 30 million people, which accounts for nearly 9.4% of the US population [1]. The number of patients with a diabetes diagnosis continues to rise at a significant pace, with an incidence rate of 1.5 million US adults per year [1]. While lifestyle modifications and medications have improved diabetic control, there is still a significant proportion of patients with uncontrolled diabetes leading to advanced complications of the disease. Prolonged uncontrolled diabetes mellitus progresses into a broad range of macrovascular complications such as heart disease and stroke secondary to accelerated atherosclerosis from glucose-induced oxidative stress [2]. Diabetes also causes microvascular complications such as diabetic retinopathy, neuropathy, and nephropathy [2]. Additionally, diabetes is the leading cause of kidney failure and new onset blindness in the United States [3]. Previous studies indicate that for each 1% reduction in hemoglobin A1c, there was a corresponding 14% reduction in myocardial infarction, 12% reduction in stroke, and a 37% reduction of microvascular complications [4]. Given the multi-organ complications associated with diabetes, it is imperative for physicians to recognize these associated complications and provide appropriate preventative care for patients with diabetes to achieve better control of their disease.

**Fig. 1:**
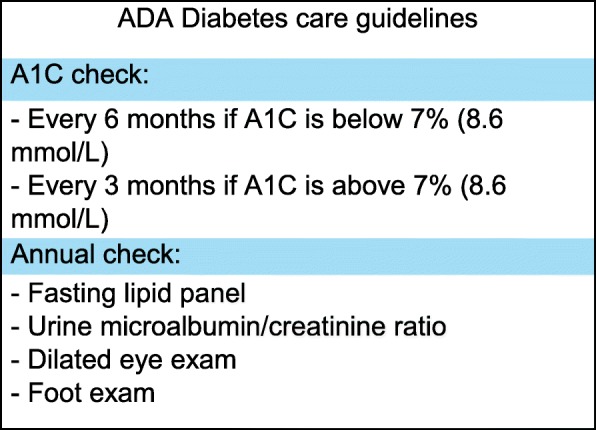
ADA Diabetes care guidelines

American Diabetes Association (ADA) sets forth annual guidelines on preventative measures that can help prevent or delay the onset of more severe complications of diabetes mellitus (Fig. 1). ADA recommends that physicians monitor HbA1c levels every 3–6 months and set a goal A1C level of under 7% (8.6 mmol/L) for appropriate control of the disease [5]. Additionally, physicians are advised to obtain lipid profiles, urine albumin/creatinine ratio, and estimated glomerular filtration rate (GFR) annually [5]. In order to monitor the retinopathy and peripheral neuropathy associated with diabetes, physicians are also recommended to perform a fundoscopic and comprehensive foot examination at annual visits [5].

**Fig. 2:**
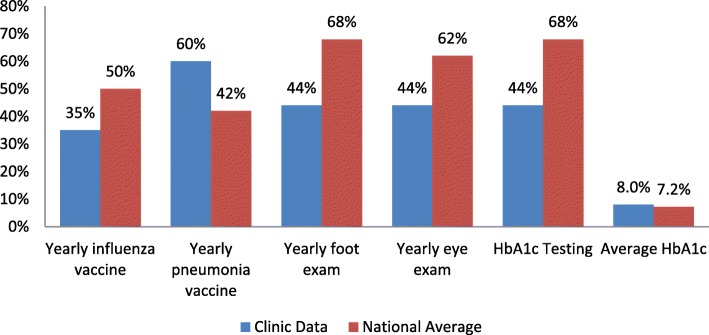
Diabetes clinic data compared with national average

As part of internal medicine residency training, medical residents are often first-line primary care providers in underserved clinics for those suffering from diabetes mellitus and thus have an important role in providing appropriate care for these patients. However, previous studies describe suboptimal care amongst residents in regards to preventative care in patients with diabetes [6]. One previous study compared diabetes care in resident clinics versus private physicians and found significant decrease in patient satisfaction (56.5% vs. 71.3%) as well as lower completion of diabetic preventative evaluations such as foot (43.3% vs. 69.1%) and eye examinations (43.8% vs. 62.8%) in resident clinics [6]. At our own clinic, prior to the onset of this study, various quality measures for diabetes care were just as suboptimal, with only 41% of the patients with diabetes receiving foot examinations and 32% receiving eye examinations (Fig. 2).

The development of quality improvement teaching and active QI projects in the residency curriculum has been increasingly emphasized in recent years. Alliance of American Medical Centers, a national network of large academic medical centers, created a national initiative to develop material for teaching quality improvement in residency curriculums and improving patient care [7]. Additionally, Accreditation Council for Graduate Medical Education (ACGME) published revisions to its common program requirements and included increased emphasis on patient safety and quality improvement as part of residency curriculums [8].

Given the emphasis on quality improvement combined with suboptimal care noted amongst resident clinics with regards to patients with diabetes, our study aims at improving the diabetic care measures in our resident clinic through implementation of quality improvement interventions.

## Methods

### Setting

Beaumont Hospital-Royal Oak is an academic medical center with the largest outpatient clinic in Southeast Michigan. The clinic has 10,000 patients actively enrolled from which approximately 10% have a diagnosis of diabetes mellitus. The clinic consists of 60 internal medicine residents and 16 medicine-pediatrics residents in different stages of training, providing a significant variability to the care delivered at the facility. The 76 residents function as primary care physicians and are divided in 8 teams, with each team having an attending physician as a team captain. All of the patients seen by the residents are discussed and separately evaluated and co-managed by the supervising physician. Additionally, the patients’ socioeconomic status can be considered below average with over 50% of the patients obtaining their care via Medicaid insurance. Given this, there is a large no-show rate for patient visits at the resident clinic [9].

### Study model

**Fig. 3:**
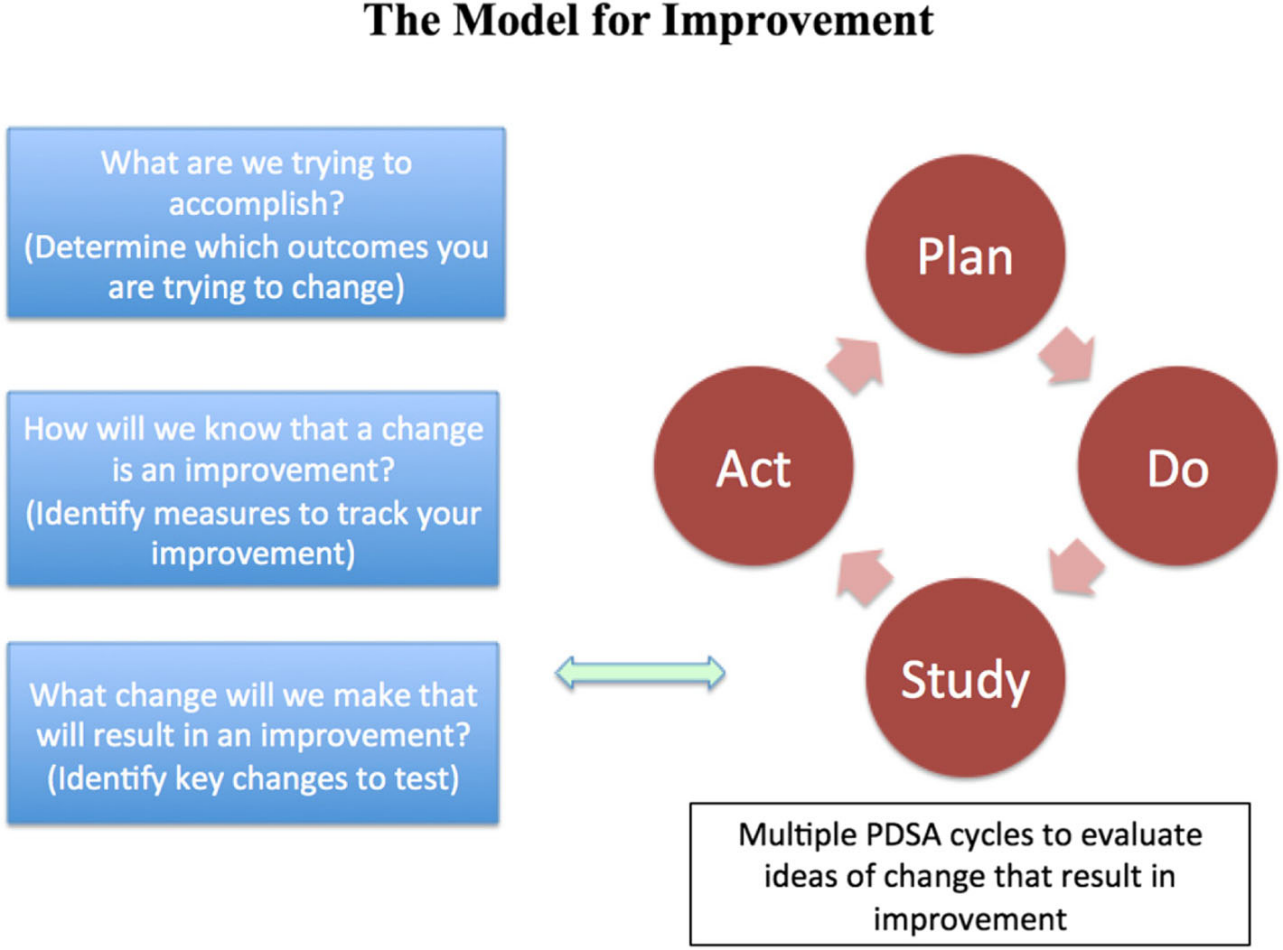
How to build a quality improvement process [10]

**Fig. 4:**
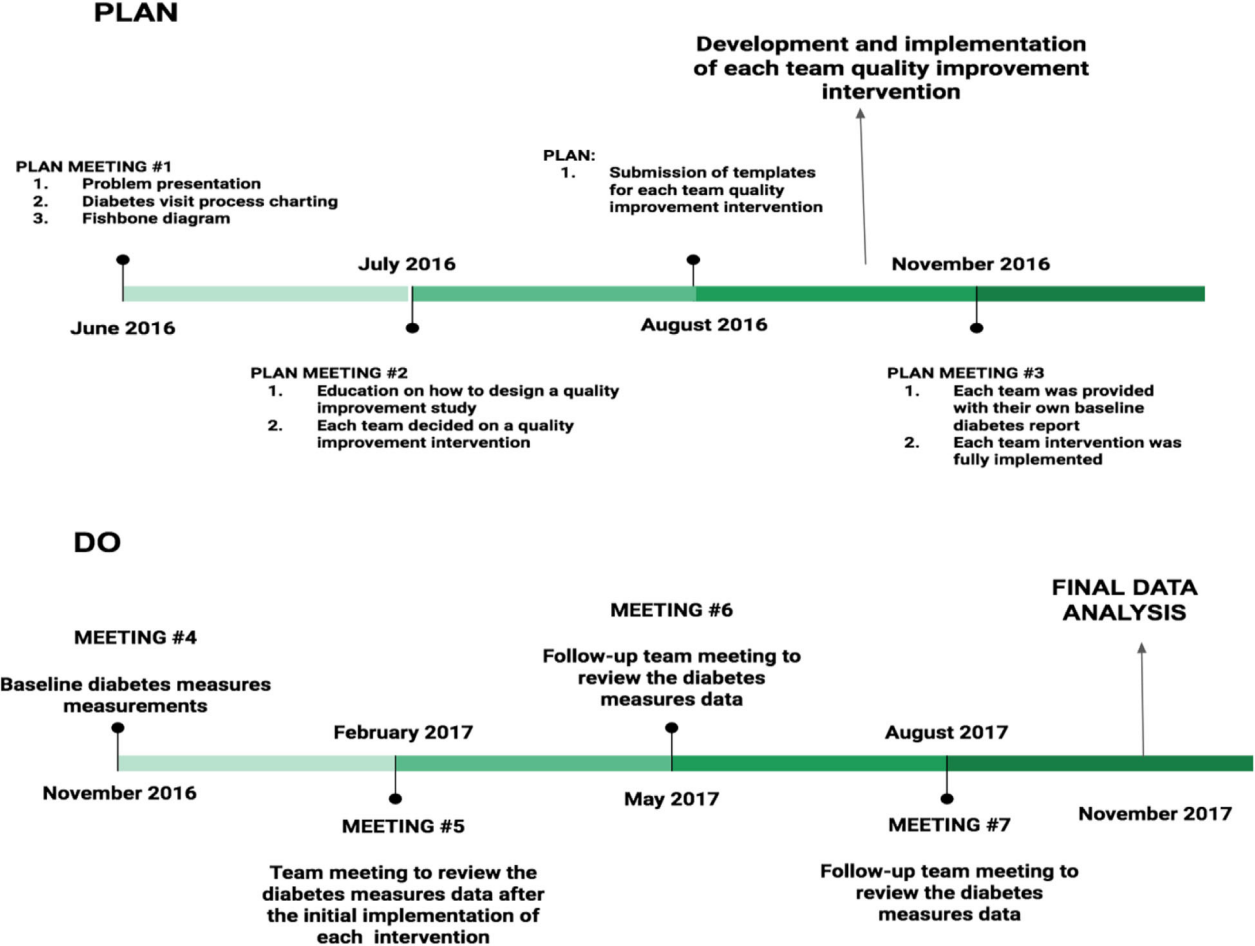
Project Timeline

This study utilized the Plan-Do-Study-Act (PDSA) framework outlined by the Institute for Healthcare Improvement (IHI) for improvement processes *(*Fig. 3*)*[10]. The study enlisted the 76 residents working in 8 different teams at the Beaumont clinic. The project was performed in two stages, the planning stage involving resident education and the intervention stage when the residents enacted an intervention to the care of their patients with diabetes (Fig. 4).

### Planning stage

**Fig. 5:**
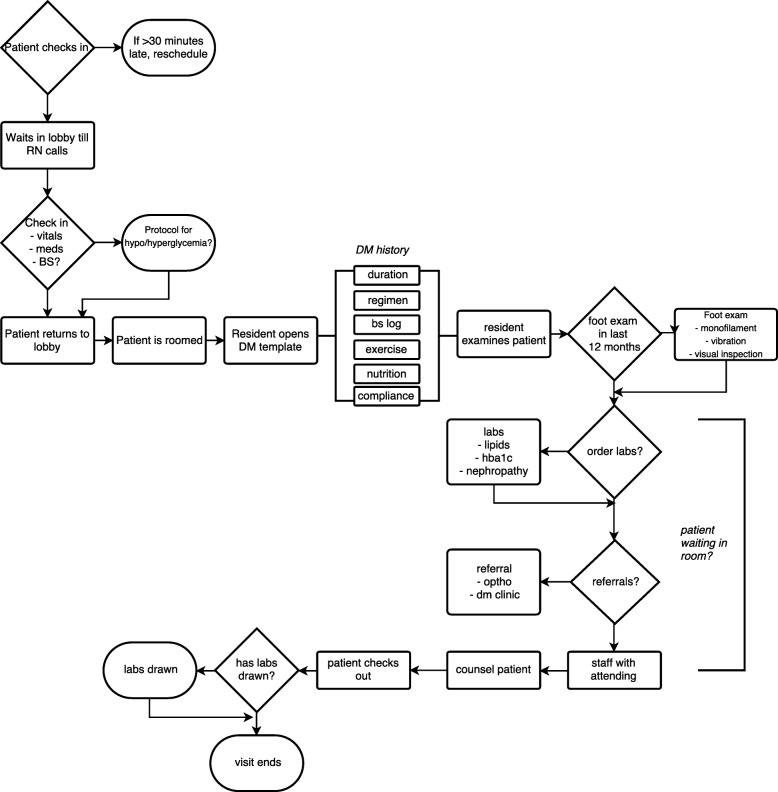
Clinic patient flow

In June 2016, the first educational meeting was scheduled, and the residents were presented information on the current diabetes outcomes in the clinic. The diabetes data was presented for the entire clinic and compared with the national benchmarks, as seen previously in Fig. 2. From June to November 2016, prior to the implementation of individual quality improvement plans, a clinic-wide optimization and standardization of patient flow (Fig. 5) as well as education regarding proper documentation of diabetes maintenance evaluations in the EMR took place (Diabetes template located in additional file 1). Additionally, two clinic-wide interventions were implemented: 1) the diabetes clinic visit template was standardized based on the ADA diabetes care guidelines and was shared with all of the residents to be used during their patients’ diabetes visits, and 2) each visit, a half page reminder with the ADA diabetes guideline for laboratory measures and the eye exam had to be filled out by the residents (Sample reminder sheet located in additional file 2). The purpose of these clinic-wide interventions was to systematically remind the residents to address these ADA guideline measures at each visit as well as to remind attendings, since the half-page documentation had to be co-signed by a faculty member.

**Fig. 6:**
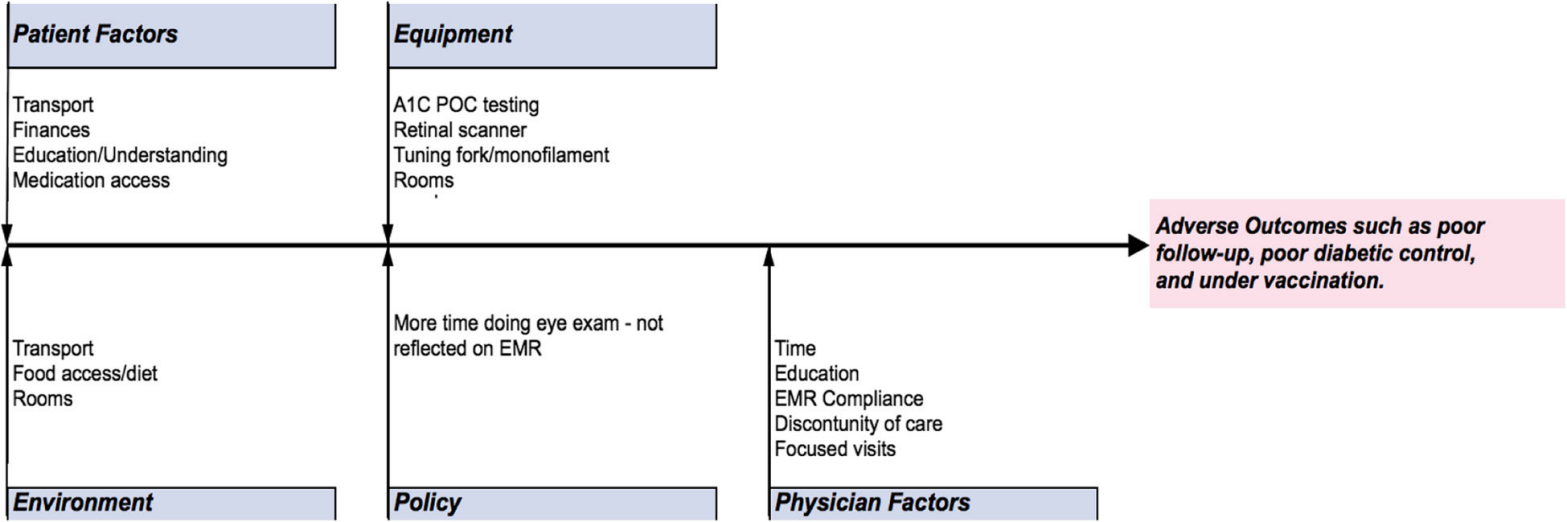
Fishbone diagram to identify opportunities for improvement

In July 2016, a second meeting took place when the residents were educated on how to design a quality improvement study (PDSA cycle) and a Fishbone diagram (Fig. 6) served as the structure to identify ways and areas to implement change. Each of the 8 teams was then advised to develop a quality improvement intervention and submit a template of the intervention by August 2016.

### Intervention stage

The impact of each resident team’s intervention was measured by comparison of the diabetes quality indicators pre and post intervention. During the timeframe of the study, the resident teams remained unchanged until July 1st 2017 at which point 3rd year residents graduated from the program and 1st year residents were added to the teams. The senior residents on each team were encouraged to meet with the new interns on their respective teams and inform them regarding their team’s intervention. However, no program-wide quality improvement educational sessions were provided for the new interns during this phase of the study period. The patients assigned to a team at the study onset remained with the same team throughout the study. In the four month period (from July 2016 to November 2016) after the implementation of the two clinic-wide interventions, each resident rotated at least once in the clinic for a one month block prior to the onset of individual quality improvement interventions.

In November 2016, we performed an EMR query through Business Objects, a software that pulls the data from our EPIC EMR, to identify patients at the resident clinic with known diabetes. On these patients, baseline data was obtained for age, gender, BMI and for each of the quality indicators (listed in Table 1) prior to the onset of team-based interventions. For patients with multiple visits to the clinic, we utilized data from the patient’s last visit prior to November 11, 2016. Each resident team was provided its baseline diabetes report. At this time, the teams that designed a quality improvement intervention plan fully implemented their interventions. The key points of each team’s intervention plan are also described (Table 2). Individual residents from each team received patient-level data with their own patients’ diabetes ADA guideline measures statistics.

**Table 1:** Outcomes evaluated in the study

Outcomes of the study	
Primary objectives	
A1C value	
Secondary objectives	
Yearly LDL testing Yearly microalbumin/creatinine ratio Yearly eye exam Yearly foot exam A1C testing at appropriate intervals

**Table 2:** QI Intervention plans for each team

Team Color	QI Intervention
Purple	1. Protected half-day during clinic month to call and schedule patients with overdue preventative diabetes care. 2. During the visit, obtain all overdue lab work, perform foot exam, and refer for eye exam if due. 3. Provide patients with information on due dates such as next visit, next eye examination, etc.
Red	1. Perform any necessary labs right after the clinic visit. 2. Team will better organize their clinic visits to ensure they know what needs to be addressed at each particular visit.
Green	1. Allow lipid panels to be performed non-fasting if patients are overdue. 2. Provide patient education on logging blood sugars appropriately.
Yellow	1. Call their patients about scheduled appointments 48 h beforehand rather than a week ahead. 2. Remind them to try obtaining their pre-visit labs a day prior to visit. 3. Attempt to input blood sugar logs into EMR to better track the values. 4. Refer more patients to high-risk diabetes clinic.
Blue	1. Patient education on diabetes complications and various weight-loss tracking applications on phones. 2. Remind patients to try obtaining their pre-visit labs a day prior to visit. 3. Make a checklist for residents so they are aware of what needs to be performed during a diabetes visit.

In February 2017, after 3 months of intervention, a meeting with the residents was scheduled to review the observed changes in quality indicators compared to the baseline data. Attendance was voluntary and no attendance data was tracked. Residents were, however, incentivized to attend and participate via free catered lunches during the meetings. The initial phase of the meeting detailed each team’s quality indices data and its comparison to the baseline report via powerpoint medium and lasted approximately 15 min. Quality indicators with significant improvement were acknowledged and areas of deficiency or no improvement were identified. The next phase of the meeting lasted 20–30 min and consisted of each team discussing their implemented plan. Points of discussion included difficulties in implementing the plan, compliance with the plan, and ideas to improve the quality indicators, especially those that showed no improvement since the baseline time period.

These meetings were repeated again at 6 months and 9 months from the onset of the intervention (May 2017 and August 2017). By November 2017, the quality improvement interventions were in place for one year and the data on the quality indicators was collected for final data analysis.

### Sample size

Sample size for this study was identified via electronic medical record (EMR) query at Beaumont hospital. The query identified all patients assigned to a resident as their primary care provider, as of November 10th 2016. Patients were identified via coding criteria for diabetes-related diagnoses within the ‘Medical history’ and ‘Problem List’ section in the EMR. After this initial query, patients who were incorrectly labeled as having diabetes, such as those with a diagnosis of pre-diabetes or borderline diabetes mellitus, were identified via individual review of patients’ diagnoses and were removed so only the patients with diabetes mellitus diagnosis were included.

**Fig. 7:**
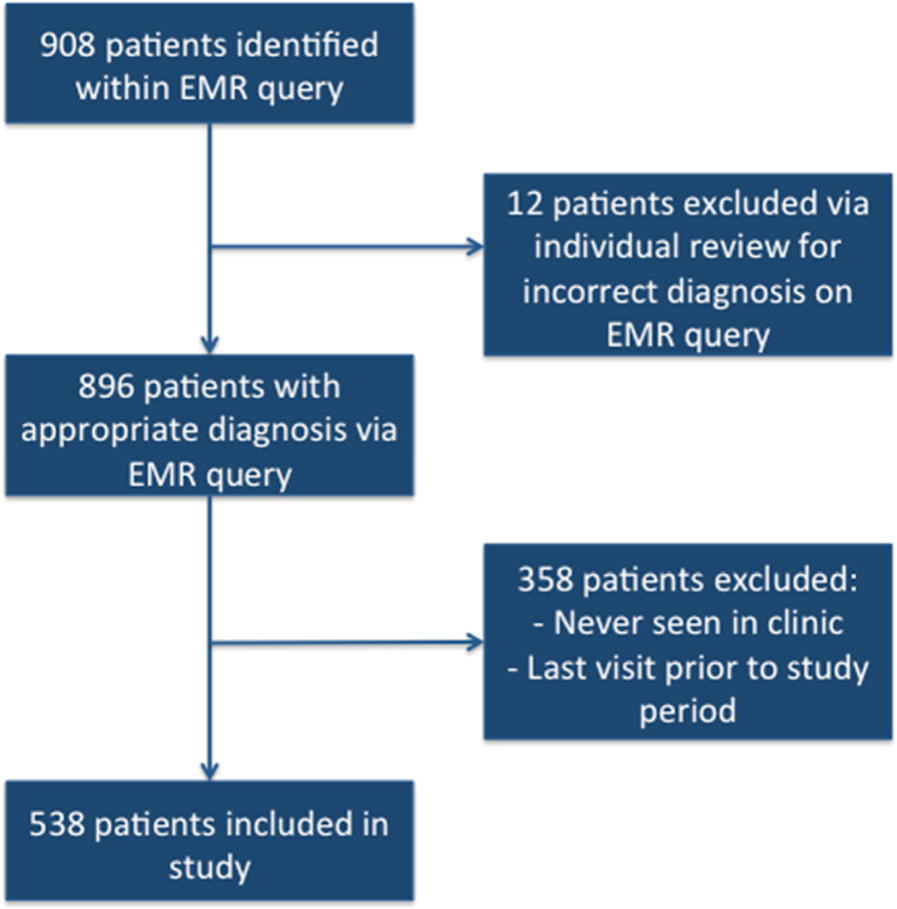
Sample size of patients included in the study

We then excluded patients who were in the query but were never seen in the resident clinic prior to the study period. Patients never seen in the clinic were included in the initial query because they had been seen within the hospital and had scheduled appointments with the clinic post-hospitalization but never actually visited the clinic. Additionally, patients who did not have at least one visit during the study period were excluded from the study since these patients did not undergo any of the interventions. The final sample size for the study was established after these exclusions (Fig. 7). The study also excluded any patients who enrolled in the resident clinic after baseline data was collected.

### Variables

The quality indicators that were observed were chosen based on ADA guidelines for key measures to monitor in patients with diabetes. The ADA has provided recommendations on frequency of testing and/or optimal levels of laboratory values in patients thus providing an opportunity to assess the effectiveness of our interventions within the context of national standards. A1C was chosen as the primary outcome given the significant reduction in complications of diabetes associated with decrease in A1C. Additionally, studies have shown that increase in patient’s perceived control of diabetes and diabetes education have had significant improvement in A1c outcomes [11, 12]. Given these effects of non-medication variables on A1c, we hypothesized that an increase in preventative examinations in our study would also reflect in an improved A1c level. As one of the secondary outcomes, hemoglobin A1C was also reported as ‘done’ or ‘overdue’ based on the status at the time of data collection. Per ADA *recommendations*, A1C was considered ‘done’ in two circumstances: if it was performed within 3 months when previous A1c was ≥ 7% or if it was performed within 6 months when previous A1c was < 7%. Foot examination, eye examination, microalbumin/creatinine ratio, and lipid panel were the remaining secondary outcomes that were also reported as ‘done’ or ‘overdue’ based on the status at the time of data collection.

### Data collection method

The current study received IRB approval from the Beaumont Research Institute prior to data collection. The data collection was performed in consultation with the Outcomes Research Director at the hospital. An electronic medical record query set as of November 10th 2016 was obtained for the following indicators: A1C value level and status (overdue or up-to-date), LDL status (overdue or up-to-date), and urine microalbumin to creatinine ratio (overdue or up-to-date). Additionally, we queried all of the patients that were overdue for a foot exam or an eye exam. Thus, baseline data collected was the last available data prior to November 10th 2016. This query process was repeated with time periods set at February 10th 2017, May 10th 2017, August 10th 2017, and November 10th 2017.

#### Statistical analysis

Descriptive statistics were reported as frequencies along with proportions for categorical variables. Means (with confidence intervals) were used to describe continuous variables. Fisher’s exact test was used to compare categorical variables. Statistical significance was considered at *p* < 0.05.

## Results

The intervention stage was performed as planned, with scheduled PDSA meetings taking place every 3 months and final data collected in November 2017, one year after the onset of intervention. The three teams that did not submit a quality improvement plan served as comparison groups while the remaining five teams were treated as intervention groups. The outcome data collected at the baseline period was compared to the same outcome data post-intervention period at one year. The change in outcome measures between the two time periods was identified as the effect of the intervention. Baseline characteristics were similar between the intervention and comparison groups (Table 3). The patients’ ages on average in intervention and comparison groups were 60.9 years and 58.9 years respectively. 54% of the patients were identified as female in both groups. Average BMI was 35.4 in intervention group and 35.9 in comparison groups.

**Table 3:** Baseline characteristics of each team’s patients

Resident Team	Number of Patients	Mean Age	Gender (% Female)	Mean BMI
Blue	76	59.6	61%	34.2
Green	65	61.9	49%	35.6
Purple	62	60.0	58%	35.2
Red	65	63.3	49%	37.5
Yellow	60	59.6	56%	34.5
Gold	62	57.5	56%	36.1
MedPeds	65	61.6	47%	36.5
Orange	83	58.9	60%	35.2
Intervention Groups	328	60.9	54%	35.4
Comparison Groups	210	58.9	54%	35.9

The primary outcome evaluated in this study was the change in A1C value in intervention and comparison groups, from before and after the QI intervention implementation. Table 4 lists the baseline and post-intervention A1C values for each individual team. As a group, the change in A1C value in the intervention group is + 0.086 versus in the comparison group, + 0.322. The difference between the intervention and comparison group was not statistically significant (*p* = 0.174).

**Table 4:** A1C values at baseline and post-intervention, intervention vs. comparison groups

	A1C at Baseline		A1C at Year 1			
	Mean A1C (%)	95% CI	Mean A1C (%)	95% CI	Change in A1C Value	95% CI
Intervention Groups						
Blue	7.92	(7.43,8.41)	8.06	(7.49, 8.63)	0.14	(−0.42, 0.70)
Green	7.64	(7.20, 8.09)	7.75	(7.34, 8.16)	0.11	(−0.31, 0.52)
Purple	8.21	(7.57, 8.85)	7.99	(7.40, 8.58)	−0.22	(− 0.77, 0.34)
Red	8.05	(7.34, 8.75)	8.18	(7.39, 8.98)	0.13	(−0.42, 0.69)
Yellow	7.73	(7.29, 8.17)	8	(7.45, 8.54)	0.27	(−0.21, 0.74)
Comparison Groups						
Gold	7.53	(7.13, 7.94)	7.71	(7.25, 8.17)	0.18	(−0.16, 0.51)
Orange	7.96	(7.40, 8.52)	8.27	(7.68, 8.86)	0.31	(−0.08, 0.70)
MedPeds	7.78	(7.27, 8.29)	8.25	(7.71, 8.79)	0.47	(0.03, 0.91)

**Fig. 8:**
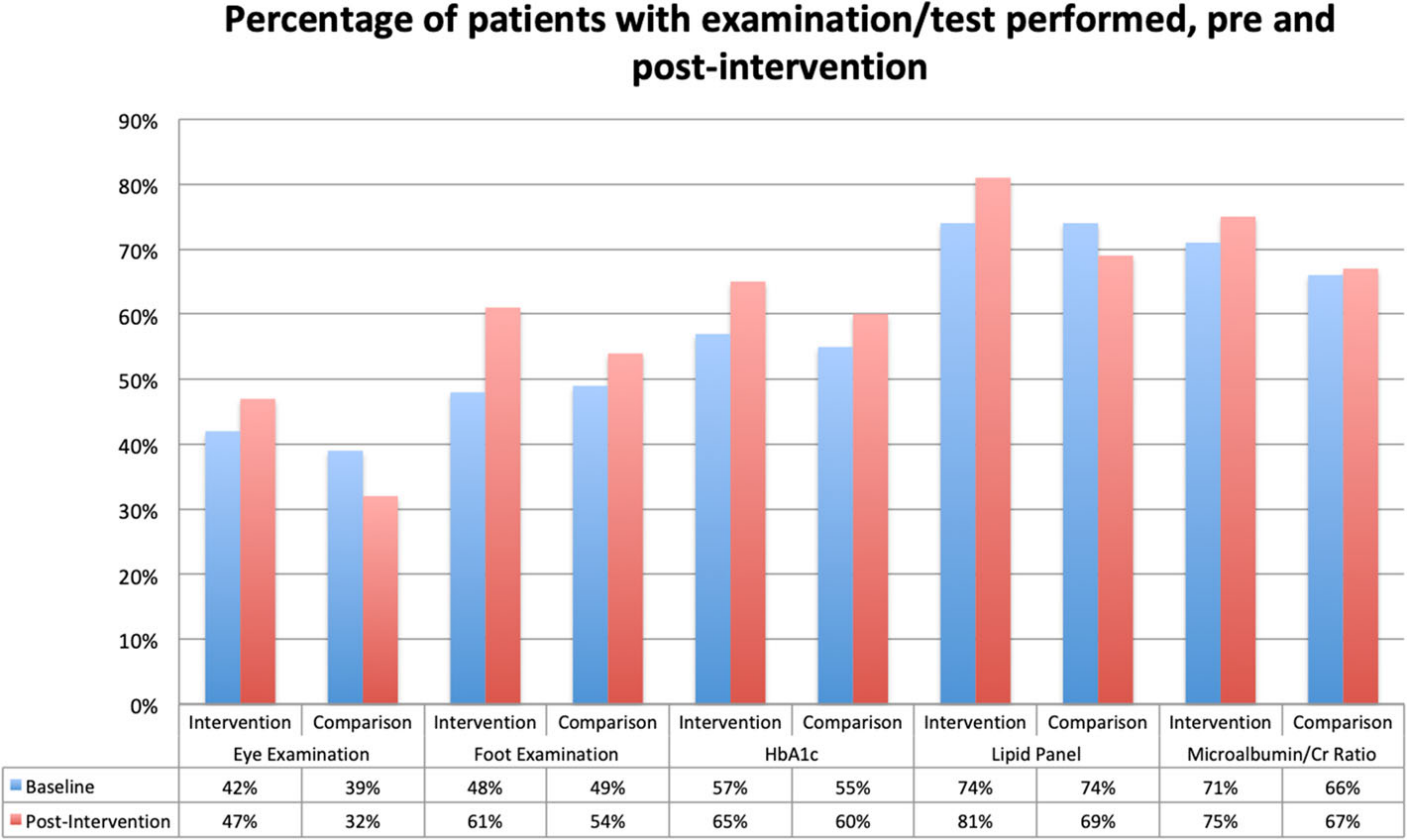
Graphical presentation of percentage of patients with examination/test performed, pre and post-intervention

The secondary outcomes evaluated in the study are listed in Table 1 above. The baseline and post-intervention data for each individual resident team in the intervention group is listed in Table 5 and presented as a change from baseline in Table 6. As a group, the changes in outcome measures were as follows: eye examinations (+ 5% in intervention vs. -7% in comparison group, *p* < 0.01), foot examinations (+ 13% vs. + 5%, *p* = 0.09), lipid panel (+ 7% vs. -5%, *p* < 0.01), micro-albumin/creatinine ratio (+ 4% vs. + 1%, *p* = 0.03), and A1C (+ 8% vs. + 5%, *p* = 0.24) (listed in Table 7 and Fig. 8). There was a statistically significant improvement in eye exams performed and lipid panel and micro albumin/creatinine ratio laboratory tests obtained. There was no statistically significant improvement in foot exams performed or percentage of A1C laboratory tests obtained.

**Table 5:** Secondary outcomes, by each resident team in intervention group (*B* baseline, *PI* post-intervention)

*Secondary Outcome*	Blue		Green		Purple		Red		Yellow		Comparison Group	
	B	PI	B	PI	B	PI	B	PI	B	PI	B	PI
Foot examination	46%	58%	59%	62%	46%	78%	57%	59%	32%	51%	44%	43%
Eye examination	39%	34%	46%	51%	42%	57%	40%	52%	41%	41%	39%	32%
Lipid panel	75%	79%	70%	76%	71%	83%	76%	88%	78%	78%	74%	69%
A1C	59%	62%	54%	57%	58%	75%	52%	69%	59%	64%	55%	60%
Microalbumin/Cr ratio	68%	75%	71%	68%	71%	78%	71%	81%	73%	76%	66%	67%

**Table 6:** Secondary outcomes, change from baseline in each intervention group (bolded = largest positive change in intervention groups)

*Secondary outcome*	Blue	Green	Purple	Red	Yellow	Comparison group
Foot examination	+ 12%	+ 3%	**+ 32%**	+ 2%	+ 19%	+ 5%
Eye examination	−5%	+ 5%	**+ 15%**	+ 12%	0%	−7%
Lipid panel	+ 4%	+ 6%	**+ 12%**	**+ 12%**	0%	−5%
A1C	+ 3%	+ 3%	**+ 17%**	**+ 17%**	+ 5%	+ 5%
Microalbumin/Cr ratio	+ 7%	−3%	+ 7%	**+ 10%**	+ 3%	+ 1%

**Table 7:** Secondary outcomes, intervention vs. comparison groups

Secondary Outcome	Group	B	PI	Change from B to PI	*P*-value
Eye Examination	Intervention	42%	47%	+ 5%	< 0.01
	Comparison	39%	32%	− 7%	
Foot Examination	Intervention	48%	61%	+ 13%	0.09
	Comparison	49%	54%	+ 5%	
A1C	Intervention	57%	65%	+ 8%	0.24
	Comparison	55%	60%	+ 5%	
Lipid Panel	Intervention	74%	81%	+ 7%	< 0.01
	Comparison	74%	69%	−5%	
Microalbumin/Cr Ratio	Intervention	71%	75%	+ 4%	0.03
	Comparison	66%	67%	+ 1%	

Percentage of patients with examination/test performed, pre and post-intervention. *B* = Baseline, *PI* = Post-Intervention. *P*-value measures the change from B to PI between intervention and comparion groups

Percentage of patients with examination/test performed, pre and post-intervention. *B* Baseline, *PI* Post-Intervention.

## Discussion

### Primary outcome

The primary outcome evaluated in this study, A1C value, did not show a statistically significant difference between intervention and comparison groups. While the lack of improvement in A1C value is paradoxical to what was anticipated at the onset of our study, similar data was seen in other studies published in literature. Specifically, the change in A1C value did not significantly improve if baseline A1C was approximately near 8% (10.2 mmol/L) [13]. Within our own resident clinic, the baseline A1C value for intervention and comparison groups was 7.9% (10.0 mmol/L) and 7.8% (9.8 mmol/L), respectively. Per Lancet’s meta-analysis of quality improvement studies evaluating A1C, this lack of improvement was also demonstrated in settings where the QI intervention involved clinician reminders and auditing, similar to the interventions used in our study*.*^*13*^ Similar findings were observed in previous studies examining the effect of quality improvement interventions on resident clinics alone. For example, in a study investigating foot examinations performed in resident clinic, the HbA1c value increased from 7.9% (10.0 mmol/L) to 8.1% (10.3 mmol/L) over the span of the QI intervention [14]. Additionally, another study performed at an internal medicine resident clinic also demonstrated a lack of decrease in A1C, irrespective of intervention or comparison group [15].

There may be several underlying reasons for the lack of improvement in A1C values. For one, the residents were unaware that A1C value was the primary outcome in our study. This was implemented to avoid bias by steering the residents away from focusing solely on the primary outcome. If our study was developed with a singular focus on A1C improvement, the results may have demonstrated improved A1C values. Another reason specific to our study is that the quality improvement interventions that were implemented in our resident clinic did not directly involve activities that decrease A1C. The interventions incorporated were primarily targeted at examinations or laboratory tests that should be performed by the clinician in the clinic, rather than patient interventions. We hypothesized that the preventative examinations may still indirectly affect A1c value but our study did not demonstrate that correlation. A possible cause for this lack of improvement is time utilization during a visit. For example, a resident who is focused on performing a foot examination may not have spent the necessary amount of time counseling on diet or medication regimen, thus mitigating the beneficial effect of a foot examination. With limited time during a clinic visit, the residents’ ability to impact both glycemic control and provide appropriate preventative care may be decreased. These results demonstrate the importance of such comprehensive diabetes care in patients with diabetes and the definite need in this patient population for parallel diabetes diet education, pharmacist education and intensive lifestyle changes [16, 17].

### Secondary outcomes

While foot examination and A1C test did not show a statistically significant improvement with the quality improvement intervention, each secondary outcome demonstrated an absolute increase in the percentage of patients who received those tests 1 year after the interventions were implemented. Compared to the national data as shown earlier in Fig. 2, the adherence rates in the clinic are still lagging behind, however there were significant improvements from the pilot study data. Specifically, the national adherence rate of foot and eye examinations are 68 and 62% respectively. In our clinic’s intervention groups, these two adherence rates improved from 48 to 61% in foot examinations performed and 42 to 47% in eye examinations performed. Similarly, with A1C testing, the intervention group improved from 57 to 65%, similar to the national rate of 68%. While these QI interventions have not completely eliminated the gap between our clinic and the national averages, the significant improvements in these rates indicate the QI interventions as potential solutions to the low adherence rates. Considering the given trend, we hope that there is further improvement in adherence rates with continued use of QI interventions.

Comparing with present literature, there were limited studies evaluating the secondary outcomes from our study. One previous project evaluating foot examinations showed similar improvements in the number of foot examinations performed post-intervention*.*^*14*^ In another research article, there was significant increase in A1C and LDL testing obtained in intervention versus the comparison groups [15]. While there were few studies reporting on these secondary outcomes, we were unable to identify any projects that showed a lack of improvement with a quality improvement intervention.

We also compared the difference in the secondary outcomes between the intervention groups. For foot examinations, there was improvement in all intervention groups compared to baseline. This may reflect the ease of performing a foot exam versus the other preventative examinations. Whereas eye examinations by ophthalmologists or laboratory tests obtained outside of clinic visit depend partly on the patients, the foot exam can be performed directly in the clinic. Thus, this may have contributed to the effectiveness of the quality improvement study on obtaining more foot examinations in patients. With regards to the eye examinations, three teams (Purple, Red, and Green) had improvements whereas two teams (Yellow and Blue) did not show improvement. Both Yellow and Blue teams identified pre-visit labs as an emphasis of their quality improvement study. Given that eye examinations/referrals are more likely to be performed post-visit, their emphasis on pre-visit testing may have lowered the effect of their QI interventions on eye examination adherence rates.

For laboratory tests such as lipid panel, A1C testing, and microalbumin/creatinine ratio, Red and Purple teams consistently had better improvements than the remaining teams. Both of these teams implemented interventions (as listed in Table 2) that involved obtaining overdue labwork right after the clinic visit. This was unique to these two teams as the other three intervention teams did not consider this in their intervention plans. Given this, there may be increased effectiveness in adhering to ADA guidelines, specifically with regards to laboratory testing, if the testing is performed right after a clinic visit. However, further studies are necessary to evaluate this intervention further.

In our study, Purple team had the largest improvements in all secondary outcomes with the exception of microalbumin/creatinine ratio (2nd highest improvement in this outcome). In retrospect, we evaluated the different interventions implemented by the resident teams to potentially identify the reason for such significant improvement in the Purple team versus the remaining teams. One particular unique intervention by the Purple team consisted of a protected one-half day block for each resident where they identify their patients who are overdue for the required examinations and subsequently call the patients to schedule appointments for these tests. This intervention may have been beneficial because residents were given just one task for the half-day, allowing them to better focus on identifying patients who are due for these examinations. The focused half-day may also have helped the residents better understand the ADA guidelines and made them more likely to perform these measures at their patients’ clinic visits. Additionally, personally speaking to the patients over the phone may indirectly have decreased the no-show rate and increased the compliance rate due to this increased communication. Given the significant improvements seen with the Purple team, expansion of this intervention for the remaining resident teams will be necessary to identify if it is a truly beneficial intervention for resident clinics. The significant time commitment associated with this intervention also necessitates further studies to evaluate whether ancillary staff can perform this intervention with similar improvements in outcomes.

The consistent improvement in adherence rates amongst comparison groups during this study was an interesting observation. One reason may be the proximity of the comparison groups to the intervention groups. Since the residents in the program work so closely together, it may be that strategies from the intervention groups were discussed with those from comparison groups and possibly implemented by individual residents. Another cause may be that comparison groups also were able to attend the discussion sessions every 3 months that evaluated the progress of QI interventions. During this time, these groups may have discussed strategies to improve their adherence rates but did not write down an official intervention strategy.

Regardless, the significant improvement in adherence rates in several secondary outcomes amongst intervention groups, especially Purple and Red teams, demonstrates that certain quality improvement interventions in resident clinic can be beneficial in better adhering to the ADA guidelines. The implementation of a quality improvement intervention not only allows for better preventative care in patients with diabetes in the resident clinics but also helps residents understand how to implement quality improvement into daily practice beyond residency and in their own clinics and hospital settings.

### Limitations

There are several limitations present in our research study. As mentioned prior, there is a large no-show rate for patient visits at the resident clinic [9]. The no show rate limits opportunities for the residents to provide the preventative care that is expected by the ADA guidelines and may lower the adherence rates compared to the national averages. Additionally, the comparison groups were in the same hospital location as intervention groups, which may have influenced sharing of intervention strategies amongst residents and may limit the pure random allocation of these groups. Furthermore, the comparison groups were not randomly assigned but were made of teams that opted not to design a QI intervention plan. This can be considered a limitation since teams that did not design a plan may consist of residents who are less motivated to engage in improving their quality measures for patients with diabetes.

Another limitation is the underreporting of tests performed. Residents may have performed foot examinations but did not report it in the EMR due to lack of time or disruption in the workflow. This could also inaccurately lower the adherence rates in the clinics. Similar phenomenon may have occurred with the laboratory tests, which could have been deferred if the patient had instead received the laboratory tests at an outside facility. If these facilities were not associated with Beaumont, those laboratory tests are not recorded in the EMR system, thus falsely lowering the adherence rates.

We also did not collect attendance data on the educational sessions prior to the study period, thus we were unable to evaluate the correlation between attendance at these sessions and the outcome measures. This can be considered a limitation since teams that had more members attend the sessions could have been more motivated to engage in their team’s quality improvement plan and have better outcomes.

Additionally, we did not address all of the variables listed in the ADA guidelines such as blood pressure and vaccinations. Thus, the effect of the QI interventions on these variables is unknown and needs to be addressed in future studies. The blood pressure was not measured in this study because of the wide variability present between different visits. The influenza vaccination was not measured because this study was started during the middle of flu vaccination season so we did not feel that our baseline and post year 1 results would be an accurate reflection of vaccination rates.

### Generalization

Beaumont Hospital – Royal Oak is an academic medical center nearby the large metropolitan city of Detroit. Our outpatient clinic has patients from both rural and urban areas of Southeast Michigan. Thus, the results of our study can be expected at other resident clinics in academic medical centers around the country. Additionally, the primary and secondary outcomes of our study were investigated from the national guidelines set by the ADA so we believe that this study can be replicated in other resident clinics and interventions can be implemented in different hospitals to a similar effect.

## Conclusions

This project was designed to evaluate the effectiveness of a quality improvement intervention on preventative diabetes care. While it did not demonstrate an improvement in A1C values, there were significant improvements in the rates of several ADA recommended examinations and laboratory tests performed by residents in intervention groups. The implementation of a quality improvement project in the resident clinic provides an opportunity to significantly improve the care of patients with diabetes and potentially avoid many of the complications associated with the disease. However, improvement in A1C values may be limited with a focus on singular aspect of diabetes care, such as preventative examinations. Rather, comprehensive care, such as diabetes pharmacy clinics that would tailor education to patient needs, has a significant role in improving A1C in patients with diabetes.

## Additional files

Additional file 1: Structure of diabetes template. This file describes the diabetes template that medical residents utilized in the resident clinic. (DOCX 295 kb)

Additional file 2: Reminder sheet with ADA diabetes guideline measures. This is a reminder sheet utilized by the medical residents in the resident clinic. (DOCX 83 kb)

## Data Availability

All pertinent data analyzed during this study are included in this published article. Any further datasets not included in this published article may be obtained from the corresponding author upon request.

## Abbreviations

ADA: American Diabetes Association
EMR: Electronic medical record
LDL: Low-density lipoprotein
QI: Quality Improvement
