# Supplementary figures and images for: A quality improvement project to increase compliance with diabetes measures in an academic outpatient setting
Source: Clin Diabetes Endocrinol. 2019 Jul 23;5:11. doi: 10.1186/s40842-019-0084-9 (PMC6651972; doi:10.1186/s40842-019-0084-9)

**Additional File 1: Structure of diabetes template**


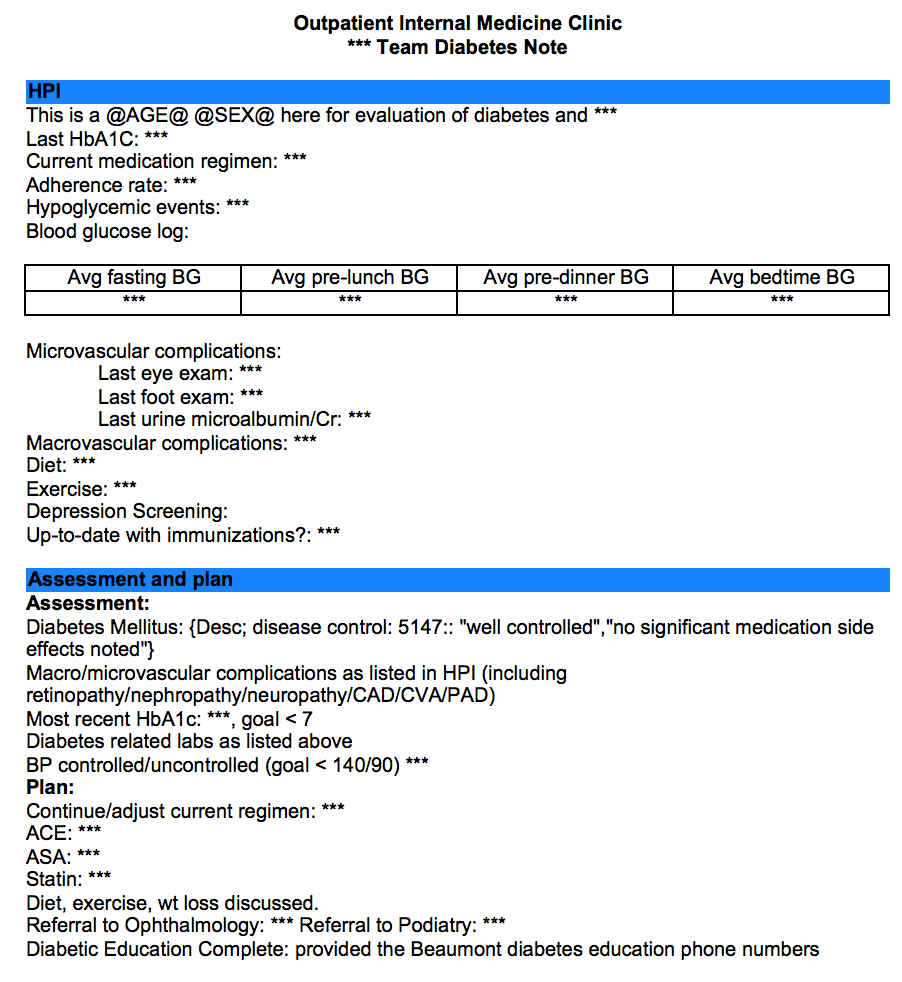

Supplement: Supplementary file 1 — Structure of diabetes template. This file describes the diabetes template that medical residents utilized in the resident clinic. (DOCX 295 kb) [file 40842_2019_84_MOESM1_ESM.docx]

**Additional File 2. Reminder sheet with core diabetes measures**


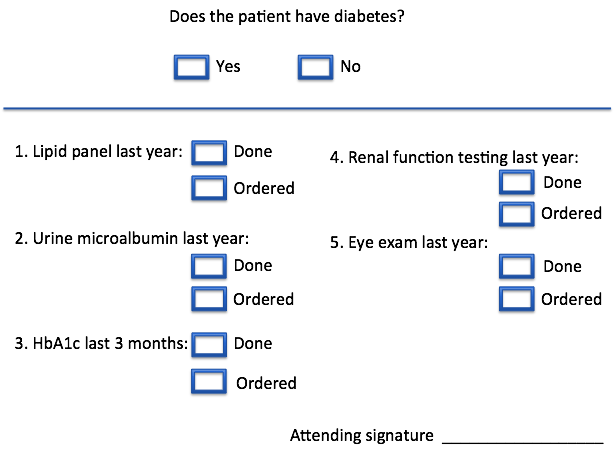

Supplement: Supplementary file 2 — Reminder sheet with ADA diabetes guideline measures. This is a reminder sheet utilized by the medical residents in the resident clinic. (DOCX 83 kb) [file 40842_2019_84_MOESM2_ESM.docx]
